# Identification and Characterization of TEX101 in Bovine Epididymal Spermatozoa

**DOI:** 10.1155/2014/573293

**Published:** 2014-04-10

**Authors:** Subir K. Nagdas, Eric L. McLean, Leeá P. Richardson, Samir Raychoudhury

**Affiliations:** ^1^Department of Chemistry and Physics, Fayetteville State University, 1200 Murchison Road, Fayetteville, NC 28301, USA; ^2^Department of Biology, Chemistry and Environmental Health, Benedict College, 1600 Harden Street, Columbia, SC 29204, USA

## Abstract

Several studies exhibit the presence of *Ricinus Communis Agglutinin I* (RCA) binding glycocalyx in mammalian spermatozoa. However, the molecular characterization of RCA binding glycocalyx in sperm membranes and its mechanism of action are poorly understood. The objective of the study was to identify and to characterize RCA binding glycoprotein of the bovine sperm plasma membranes (PM). Lectin blots of caput and cauda sperm PM revealed a 38 kDa polypeptide exhibiting the highest affinity to RCA among the several major RCA binding polypeptides. The 38 kDa RCA binding polypeptide of cauda sperm PM was purified and exhibited a charge train of three distinct spots with isoelectric points (pH 5.3 and 5.8). Proteomic identification yielded ten peptides that matched the sequence of Testis Expressed 101 protein (TEX101). Western blots data revealed that bovine sperm TEX101 is present in both testicular and epididymal sperm PM fractions. The native TEX101 polypeptide contains *~*17 kDa N-linked oligosaccharides and the polypeptide is anchored to sperm membrane via a glycosylphosphatidylinositol lipid linkage. Immunofluorescence staining of sperm with anti-TEX101 demonstrated that the polypeptide is localized at the head of cauda sperm. Our biochemical results provide evidence on the presence of TEX101 in bovine epididymal sperm plasma membranes and may have a potential role in sperm-egg interaction.

## 1. Introduction

Spermatozoa leave the testis as morphologically differentiated cells, but they require posttesticular maturation in the epididymis to develop forward both motility and fertilizing ability [1]. Mammalian sperm undergo several maturational changes during their transport through the epididymal duct, including changes in membrane lipids and proteins, morphological changes in acrosome, and cross-linking of nuclear protamines and proteins of the outer dense fiber and fibrous sheath [1, 2]. Although spermatozoa are incapable of protein synthesis, their plasma membrane proteins undergo several compositional changes via the addition of new components to the sperm surface, the unmasking or modification of preexisting sperm-surface moieties, or the loss of sperm-surface components [3].

Several studies reveal that the mammalian sperm plasma membrane surface is coated with various glycoproteins [4–6]. Sperm surface glycoproteins are thought to induce sperm maturation and fertilizing capacity in the epididymis [5, 7]. The extent to which these surface glycocalyx components are altered varies from species to species and differs in each epididymal region [8]. A hallmark feature of the mammalian spermatozoon is its highly polarized architecture, which is exhibited both in the restricted distribution of specific organelles and in the partitioning of its plasma membrane into domains of distinct composition and function [1, 8, 9]. The importance of this polarity is emphasized during discrete fertilization steps, which require the participation of selected organelles and membrane domains. The mechanisms utilized by sperm to undergo the maturational process are still not completely understood; however, the epididymis does provide sperm with an environment essential for the acquisition of motility and fertilizing ability in vivo [1]. Lectins are a class of proteins that can be used to analyze density and distribution variations of exposed saccharides in the sperm's plasma membrane [5]. Lectins of differing saccharide specificity have been shown to interact with the plasma membrane of sperm of different species [5, 10–13]. Several investigators also reported the RCA1 binding glycocalyx in mammalian spermatozoa [4, 5, 14]. A 41 kDa protein in boar epididymal sperm [4] and a 31 kDa protein in rat distal caput and cauda sperm plasma membranes exhibit strong specificity to RCA1 [14]. Using FITC conjugated RCA1, it has also been shown that RCA1 binding polypeptides were present in both head and tail of ram caput epididymal spermatozoa, whereas in corpus and cauda epididymal spermatozoa the RCA1 binding proteins are only present in head [5]. However, the molecular characterization of RCA binding glycocalyx in sperm membranes and their mechanism of action are poorly understood. The objective of the present study was to identify and to characterize* Ricinus Communis Agglutinin I* (RCA) binding glycoprotein of bovine epididymal sperm plasma membranes. We identified a 38 kDa RCA binding polypeptide present in both caput and cauda sperm plasma membrane fractions and proteomic identification revealed that the 38 kDa polypeptide is a Testis Expressed 101 protein (TEX101) (Bos Taurus). We propose that the TEX101 polypeptide may play an important role in sperm-egg interaction.

## 2. Methods

### 2.1. Sperm Preparation

Bovine epididymides were purchased from Martin's Abattoir in Godwin, North Carolina. Epididymides were stored at 4°C during transit and utilized for sperm preparation within 30 minutes of retrieval. To facilitate sperm release, caput and cauda epididymal regions were removed from the organ, minced, and incubated for 5 minutes at 37°C in Hank's balanced saline solution, pH 7.4, containing 5 mM HEPES, 2 mM benzamidine, and 0.05% sodium azide. To evaluate sperm motility, sperm were examined by phase-contrast microscopy. Sperm suspensions were centrifuged at 100 ×g for 1 minute to sediment epididymal tubule fragments. Supernatants were centrifuged at 1500 ×g for 10 minutes at 4°C using an Eppendorf Centrifuge 5403 (Brinkman Instruments, Inc, Westbury, New York). Pellets were washed 3 times by resuspension in Hank's balanced saline solution, as stated above, following which they were resuspended in a Tris-saline-protease inhibitor solution (TNI) containing 150 mM NaCl, 25 mM Tris-HCl, (pH 7.5), 2 mM benzamidine, 1 *μ*g/mL leupeptin, 1 *μ*g/mL pepstatin, and 0.05% sodium azide and centrifuged at 1500 ×g for 10 minutes at 4°C. Resulting pellets were washed 2 more times in TNI as stated above [15].

### 2.2. Isolation of Fluid/Particulate Fractions

Caput and cauda epididymides were dissected and minced in TNI at 37°C. Sperm suspensions were centrifuged at 100 ×g for 1 minute to sediment any tissue fragments that may be present within the sample. The resulting supernatants were recentrifuged at 1500 ×g for 10 minutes at 4°C; sperm pellets and supernatants were collected. Resulting supernatant fluids were again centrifuged at 100,000 ×g for 20 minutes to obtain caput and cauda epididymal luminal fluids [16].

### 2.3. Isolation of Testicular Spermatozoa

Bovine testicular spermatozoa were prepared following the method of NagDas et al. [17]. One bovine testis was minced in 30 mL of calcium-free Tyrode medium, incubated for 5 minutes at 37°C, and centrifuged at 100 ×g for 5 minutes. The supernatant solution was mixed with 120 mL 65% Percoll in Tyrode medium and then centrifuged at 23,000 rpm for 15 minutes in a 70Ti rotor. Two bands were present after centrifugation. The lower band, containing spermatozoa and some red blood cells, was diluted with Tyrode medium, layered on a discontinuous Ficoll density gradient containing 2 mL each of 10% (W/V), 20% (W/V), and 40% (W/V) Ficoll in Tyrode medium, and centrifuged at 1500 ×g for 10 minutes at 4°C. The testicular spermatozoa at the 10%–20% interface were examined for acrosomal integrity by phase contrast microscopy, diluted with Tyrode medium, and pelleted by centrifugation at 1500 ×g for 10 minutes at 4°C.

### 2.4. Isolation of Sperm Plasma Membranes

Testicular, caput, and cauda isolated sperm pellets were resuspended in TNI, disrupted by nitrogen cavitation at 400 psi for 10 minutes and pelleted by centrifugation at 1500 ×g for 10 minutes at 4°C [17]. Aliquots of the resulting supernatants were layered on discontinuous sucrose gradients composed of 20% and 50% sucrose solutions. Gradients were centrifuged at 25,000 rpm for 60 minutes in a Beckman SW40 rotor (Beckman Instruments, Palo Alto, California). Resulting plasma membrane bands (at the 20%/50% interface) were collected and diluted with TNI and centrifuged at 30,000 rpm for 35 minutes at 4°C in a Beckman SW40 rotor. Resulting plasma membrane pellets were resuspended in TNI.

### 2.5. Preparation of Cell Fractions for Western Blot Analysis

To prepare soluble and particulate fractions, testicular spermatozoa were suspended in TNI and sonicated four times for 10 seconds at the medium power setting. The sonicated suspension was centrifuged at 100,000 ×g for 30 minutes at 4°C in a Beckman SW40 rotor. A detergent-soluble fraction of caput and cauda epididymal spermatozoa was prepared by extraction with 0.1% Triton X-100 in TNI for 1 hour at 4°C followed by centrifugation at 12,000 ×g for 10 minutes. The supernatant fractions of both caput and cauda epididymal spermatozoa were used for lectin blot analysis.

Cauda sperm plasma membrane was extracted in high salt (0.5 M NaCl) in TNI for 1 hour at 4°C followed by centrifugation at 30,000 rpm for 35 minutes at 4°C in a Beckman SW40 rotor. The supernatant was collected and the pellet was suspended in TNI. The volume of both pellet and supernatant fractions were made equal to that used in the initial high salt extraction step.

### 2.6. Purification of the 38 kDa RCA Binding Polypeptide

Bovine cauda sperm plasma membrane samples were extracted in 0.1% Triton X-100 for 1 hour at 4°C and then centrifuged at 100,000 ×g for 30 minutes at 4°C. The resulting supernatant was dialyzed overnight in a 25 mM Tris-HCl pH 7.5 (elution buffer). The dialyzed sample was applied to a DEAE-Sephacel column previously equilibrated with elution buffer. To remove unbound polypeptides, the column was washed with elution buffer. Bound polypeptides were eluted with a step gradient of 0.1 M NaCl and 0.4 M NaCl in elution buffer. The 38 kDa RCA binding protein containing fraction was dialyzed against lectin buffer (25 mM Tris-HCl, pH 7.5, 150 mM NaCl, 2 mM MgCl_2_, 2 mM MnCl_2_, and 2 mM CaCl_2_). The dialyzed fraction was applied to a RCA-agarose column. The column was washed with lectin buffer and the bound protein was eluted with 0.2 M D-galactose in lectin buffer. The eluted fraction was dialyzed against water and lyophilized to powder for SDS-PAGE and 2D-PAGE analyses.

### 2.7. Proteomic Analysis

Proteomic identification of 38 kDa RCA binding polypeptide was performed at the Mass Spectrometry Facility of UNC School of Medicine Proteomic Center, Chapel Hill, NC. The 38 kDa spots from 2D-PAGE were subjected to MALDI-TOF-TOF analysis to obtain internal amino acid sequences of several tryptic peptides. Derived peptide sequences were analyzed in the National Center for Biotechnology Information (NCBI) database for the determination of potential functional motifs.

### 2.8. SDS-PAGE, Western Blotting, and Lectin Staining

SDS-PAGE was performed on 12.5% or 15% acrylamide gels [18]. Polypeptide bands were visualized either by Coomassie Brilliant blue R (CBB) [19] or silver [20] staining. Western blots were prepared on PVDF membranes [21]. Two-dimensional PAGE (2D-PAGE) was performed using a Bio-Rad precast immobilized pH (3–10) gradient gel ready for isoelectric focusing (IEF). Lectin blot analyses were done as previously described [14]. Immunoblots were briefly rinsed with TBS buffer containing 10 mM Tris-HCl, pH 7.5, 100 mM NaCl, and 0.1% Tween-20 (TBS-0.1% Tween-20) prior to blocking. To prevent nonspecific binding, membranes were blocked in a solution containing TBS-0.1% Tween-20 and 1% Bovine Serum Albumin (BSA) for 1 hour at room temperature. Blots were incubated with TEX101 polyclonal antibody (1 : 1000 dilution; Santa Cruz Biotechnology Inc., Santa Cruz, CA, USA) in blocking solution as described above for 1 hour at room temperature. To show the specificity of commercially available TEX101 antibody, immunoblots were performed using TEX101 antibody that had been preincubated with TEX101 (S-13) peptide in a 1 : 1 ratio (Santa Cruz Biotechnology Inc., Santa Cruz, CA, USA) for 1 hour at room temperature. Membranes were washed three times (3 minutes per wash) with TBS-0.1% Tween-20 and incubated with an affinity purified horseradish peroxidase-conjugated secondary antibody (1 : 2000 dilution). Immunoreactive protein bands were visualized either by color development with diaminobenzidine and H_2_O_2_ or by chemiluminescence using Super Signal (Pierce) and detection on Kodak BioMax film.

### 2.9. Immunocytochemistry

For immunofluorescence microscopy of intact cauda epididymal sperm, prepared as described above, sperm were fixed at 4°C in 4% formaldehyde in 0.1 M sodium phosphate buffer, with pH 7.6, for at least 30 minutes, attached to poly-L-Lysine coated coverslips, washed with PBS, and permeabilized by incubation for 10 minutes in −20°C acetone. After three rinses in PBS, nonspecific protein binding sites were blocked in PBS containing 0.1% Tween-20 and 2.5% BSA (blocking solution). Coverslips were then incubated with anti-TEX101 antibody in blocking solution, washed, and incubated with FITC-conjugated donkey anti-goat IgG (Santa Cruz Biotechnology Inc., Santa Cruz, CA, USA) in blocking solution. Coverslips were washed with PBS and the cells were examined by phase contrast and epifluorescence microscopy.

### 2.10. Enzymatic Digestion of Cauda Sperm Plasma Membrane

The cauda sperm plasma membrane was treated with N-glycanase (New England Biolabs, Ipswich, MA, USA), an endoenzyme that cleaves all N-linked oligosaccharide chains from glycoproteins for 24 hours at 37°C [22]. For phosphatidylinositol-specific phospholipase C (PIPLC) treatment, cauda sperm plasma membranes were washed in phosphate-buffered saline (PBS) and then incubated in PBS containing 2.0 U/mL PIPLC (Sigma-Aldrich) at 22°C for 30 minutes [23].

## 3. Results

### 3.1. Identification of *Ricinus Communis Agglutinin* (RCA) Binding Glycoproteins in Caput and Cauda Epididymal Spermatozoa and Epididymal Fluid

**Figure 1:**
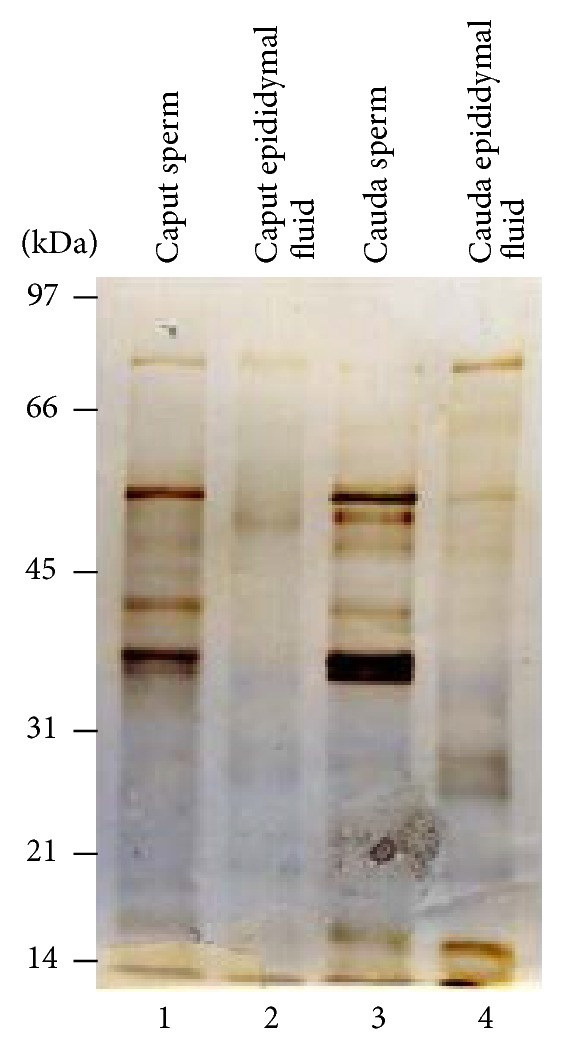
*Ricinus Communis Agglutinin I* (RCA) binding glycoprotein pattern of bovine caput and cauda epididymal spermatozoa and epididymal fluid. Western blots of Triton X-100-soluble fraction of bovine caput (lane 1) and cauda (lane 3) epididymal spermatozoa and caput (lane 2) and cauda (lane 4) epididymal fluid stained with biotinylated RCA exhibited the presence of an array of intense RCA binding polypeptides in the approximate molecular weight range of 55–38 kDa in addition to several faint RCA binding polypeptides. Among the several RCA stained bands, a doublet of 37-38 kDa polypeptides was present in both caput (lane 1) and cauda (lane 3) sperm. Caput (lane 2) and cauda (lane 4) epididymal fluid displayed several faint RCA stained bands. The amount of proteins loaded in each lane was 20 *μ*g.

To identify the maturation-dependent RCA binding glycoproteins pattern in bovine epididymal spermatozoa and epididymal fluid, lectin blots of detergent-soluble caput sperm (Figure 1, lane 1) and cauda sperm (Figure 1, lane 3) fractions and the isolated epididymal fluid from the caput (Figure 1, lane 2) and cauda (Figure 1, lane 4) epididymis were stained with biotinylated RCA. Bovine caput (Figure 1, lane 1) and cauda (Figure 1, lane 3) sperm Triton X-100 extracted supernatant fractions revealed an array of intense RCA binding polypeptides in the approximate molecular weight range of 55–38 kDa in addition to several faint RCA binding polypeptides. Among the several RCA stained bands, a doublet of 37-38 kDa polypeptides was present in both caput and cauda sperm. On the contrary, caput (Figure 1, lane 2) and cauda (Figure 1, lane 4) epididymal fluid displayed several faint RCA stained bands and did not reveal any significantly diverse banding patterns. The specificity of RCA interaction was examined by preincubation of 0.2 M D-galactose with biotinylated RCA. D-galactose resulted in marked inhibition of RCA binding. No RCA binding polypeptides were found (data is not shown), demonstrating the specificity of the RCA glycoprotein staining. This study suggests that the 37-38 kDa glycopolypeptides were the major RCA binding polypeptides among the other polypeptides present in both detergent-soluble fractions of caput and cauda sperm.

### 3.2. RCA Binding Glycocalyx Pattern of Bovine Caput and Cauda Plasma Membrane Fractions

**Figure 2:**
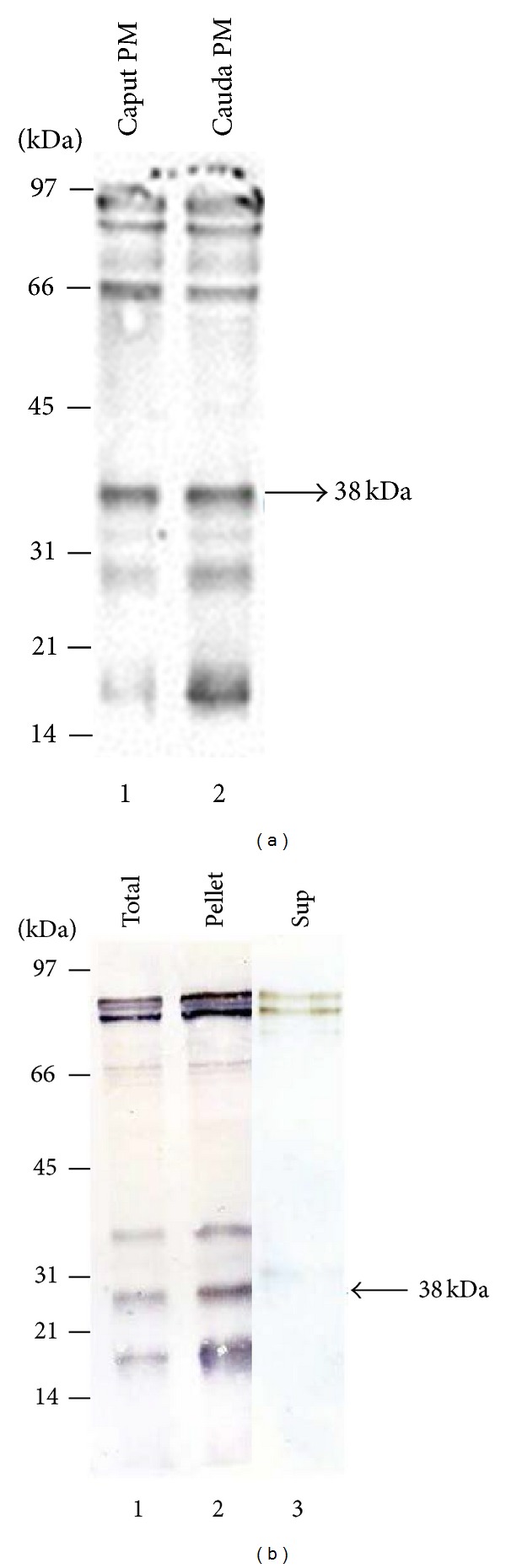
(a) RCA binding glycoproteins in plasma membranes (PM) of caput and cauda epididymal spermatozoa. Both caput (lane 1) and cauda (lane 2) plasma membrane fractions exhibited several major RCA binding polypeptides (94, 90, 66, 38, 29, and 18 kDa) in addition to several minor RCA binding polypeptides. The 38 kDa polypeptide revealed the highest affinity to RCA among the several major RCA binding polypeptides. Each lane contained 10 *μ*g protein. (b) The salt-dependent dissociation of 38 kDa RCA binding polypeptide of cauda sperm plasma membranes. Lectin blot stained with biotinylated RCA showing total plasma membranes (lane 1) and the high salt extracted pellet (lane 2) and the high salt soluble (lane 3) fractions obtained from cauda sperm plasma membranes. The 38 kDa RCA binding polypeptide is only associated with the high salt extracted pellet.

We examined the differences among RCA binding sperm-surface glycoproteins in caput and cauda plasma membrane fractions. Western blots of the enriched plasma membrane fractions of caput (Figure 2(a), lane 1) and cauda (Figure 2(a), lane 2) spermatozoa stained with biotinylated RCA. Both caput (Figure 2(a), lane 1) and cauda (Figure 2(a), lane 2) plasma membrane fractions showed several major RCA binding polypeptides (94, 90, 66, 38, 29, and 18 kDa) in addition to several minor RCA binding polypeptides. We did not observe any noticeably different RCA binding polypeptides pattern both in caput and cauda sperm plasma fractions. This study reveals that the 38 kDa polypeptide exhibits the highest affinity to RCA among the several major RCA binding polypeptides both in caput and cauda sperm plasma membrane fractions. The following studies were focused on the identification and the characterization of 38 kDa RCA binding polypeptide from cauda sperm plasma membrane fraction.

We next examined the solubility pattern of 38 kDa RCA binding polypeptide of cauda sperm plasma membrane fraction in high salt (0.5 M NaCl). Lectin blot of the total (Figure 2(b), lane 1), the pellet (Figure 2(b), lane 2), and the high salt soluble fraction (Figure 2(b), lane 3) stained with biotinylated RCA showed the presence of all major RCA binding polypeptides (94, 90, 66, 38, 29, and 18 kDa) in the high salt extracted particulate fraction (Figure 2(b), lane 2) whereas a minor fraction of 94 and 90 kDa RCA binding polypeptides were present into the soluble fraction (Figure 2(b), lane 3). This study suggests that the 38 kDa RCA binding polypeptide is an integral component of bovine cauda sperm plasma membranes.

### 3.3. Purification of 38 kDa RCA Binding Polypeptide from Bovine Cauda Sperm Plasma Membrane Fraction and Proteomic Analysis of 38 kDa RCA Binding Polypeptide

**Figure 3:**
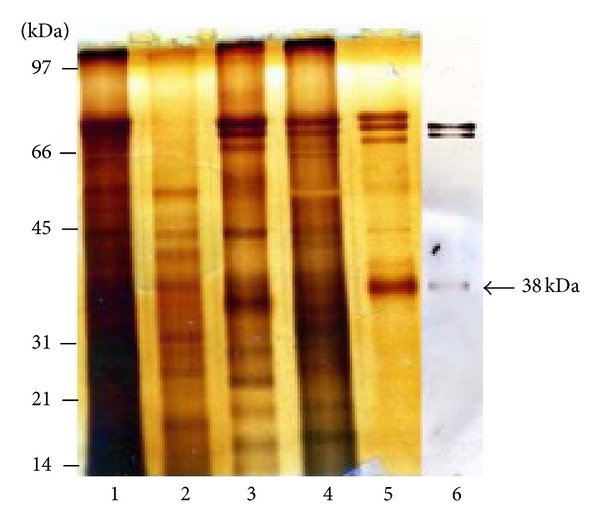
Silver stained SDS-polyacrylamide gel electrophoresis demonstrating the purification of 38 kDa RCA binding polypeptide. Lane 1 shows the complex polypeptide pattern present in the detergent soluble fraction of cauda sperm plasma membranes. Lane 2: unbound fraction of DEAE-Sepharose column. Lane 3: 0.1 M NaCl eluted fraction of DEAE-Sepharose column; 38 kDa RCA binding polypeptide was present in this fraction. This fraction was further purified by affinity chromatography on RCA-agarose. Lane 4: unbound fraction of RCA-agarose column. Lane 5: 0.2 M D-galactose eluted fraction of RCA-agarose column. Lectin blot analysis of 0.2 M D-galactose eluted fraction of RCA-agarose column exhibits the presence of the 38 kDa RCA binding polypeptide (lane 6).

**Figure 4:**
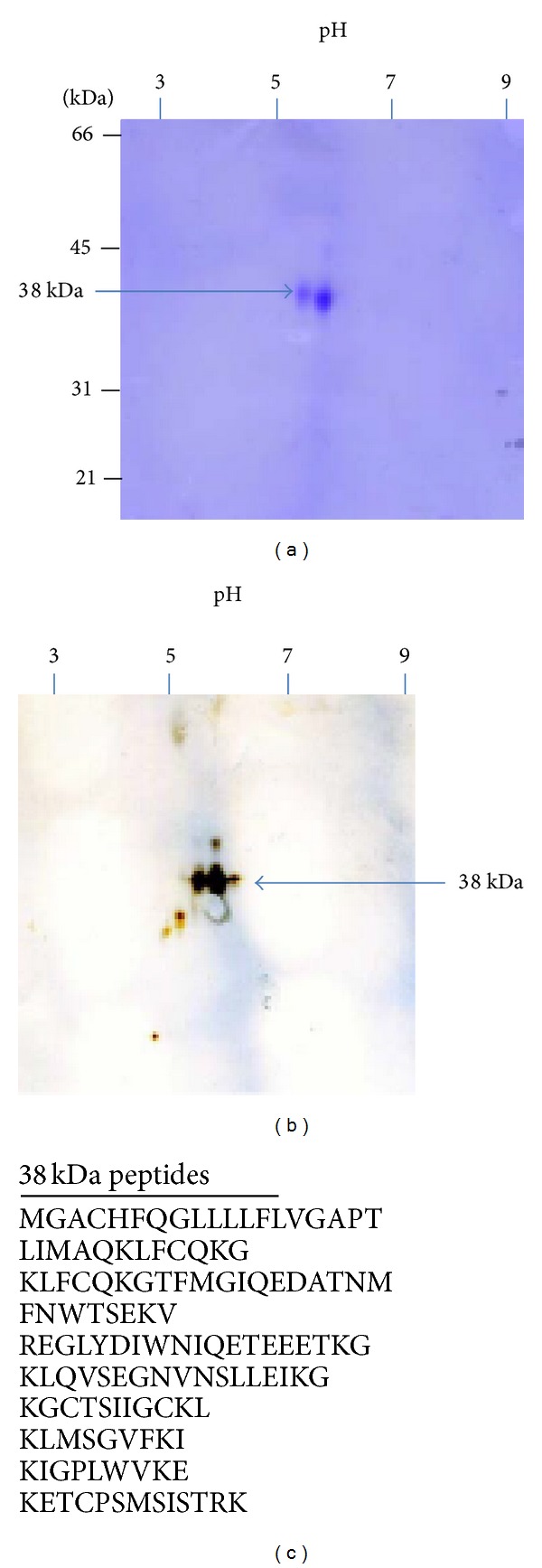
(a) and (b) purified fraction of RCA-agarose column fractionated by two-dimensional polyacrylamide gel electrophoresis stained with Coomassie Brilliant blue R (a) exhibited two distinct spots (38 kDa) with pI of 5.4–5.8. Both spots were excised and identified by proteomic analysis. Western blot of same fraction stained with biotinylated RCA (b) reveals three isoelectric variants of 38 kDa polypeptide in the pH range 5.3 to 5.8. (c) Tryptic peptides of the 38 kDa polypeptide identified by MALDI-TOF-TOF proteomic analysis.

To purify the 38 kDa RCA binding polypeptide, the Triton X-100 soluble fraction of bovine cauda sperm plasma membrane (Figure 3, lane1) was first fractionated by ion exchange chromatography on DEAE-Sepharose. The 38 kDa RCA binding polypeptide was detected in elution buffer containing 0.1 M NaCl. By ion exchange chromatography, we eliminated several polypeptides as it was observed in the unbound fraction of DEAE-Sepharose column (Figure 3, lane 2) stained with silver. The silver stained gel of 0.1 M NaCl eluted fraction of DEAE-Sepharose column (Figure 3, lane 3) revealed the presence of an array of polypeptides where the 38 kDa RCA binding polypeptide is one of them. To purify the 38 kDa RCA binding polypeptide from this 0.1 M NaCl eluted sample of DEAE column, an affinity chromatography on RCA-agarose was performed. The 38 kDa RCA binding polypeptide was eluted from the RCA-agarose column in elution buffer containing 0.2 M D-galactose (Figure 3, lane 5) and was well separated from other polypeptides (Figure 3, lane 4) as it was detected by silver stained gel. Lectin blot analysis of 0.2 M D-galactose eluted fraction stained with biotinylated RCA exhibited the presence of a 38 kDa RCA binding polypeptide (Figure 3, lane 6). The galactose eluted fraction was dialyzed against distilled water and lyophilized to powder. Several batches of the lyophilized fractions were analyzed by two-dimensional PAGE and stained with Coomassie Brilliant blue R. Two distinct stained spots (approximate molecular weight 38 kDa) with isoelectric points ranging between pH 5.4 and 5.8 were exhibited in two-dimensional PAGE (Figure 4(a)). Both spots were excised and identified by proteomic analysis. To determine if 38 kDa RCA binding polypeptide was comprised of charge variant isoforms, the lyophilized fraction was separated by two-dimensional PAGE and subjected to lectin blot analysis. Western blot stained with biotinylated RCA exhibited a charge train of three distinct spots of 38 kDa RCA binding polypeptide with isoelectric points ranging between pH 5.3 and 5.8 (Figure 4(b)). Proteomic identification of the 38 kDa RCA binding polypeptide by MALDI-TOF-TOF analysis yielded 10 peptides (Figure 4(c)) that matched the NCBI database sequence of Testis Expressed 101 protein (TEX101) (Bos Taurus).

### 3.4. Membrane Anchoring of TEX101 in Spermatozoa

**Figure 5:**
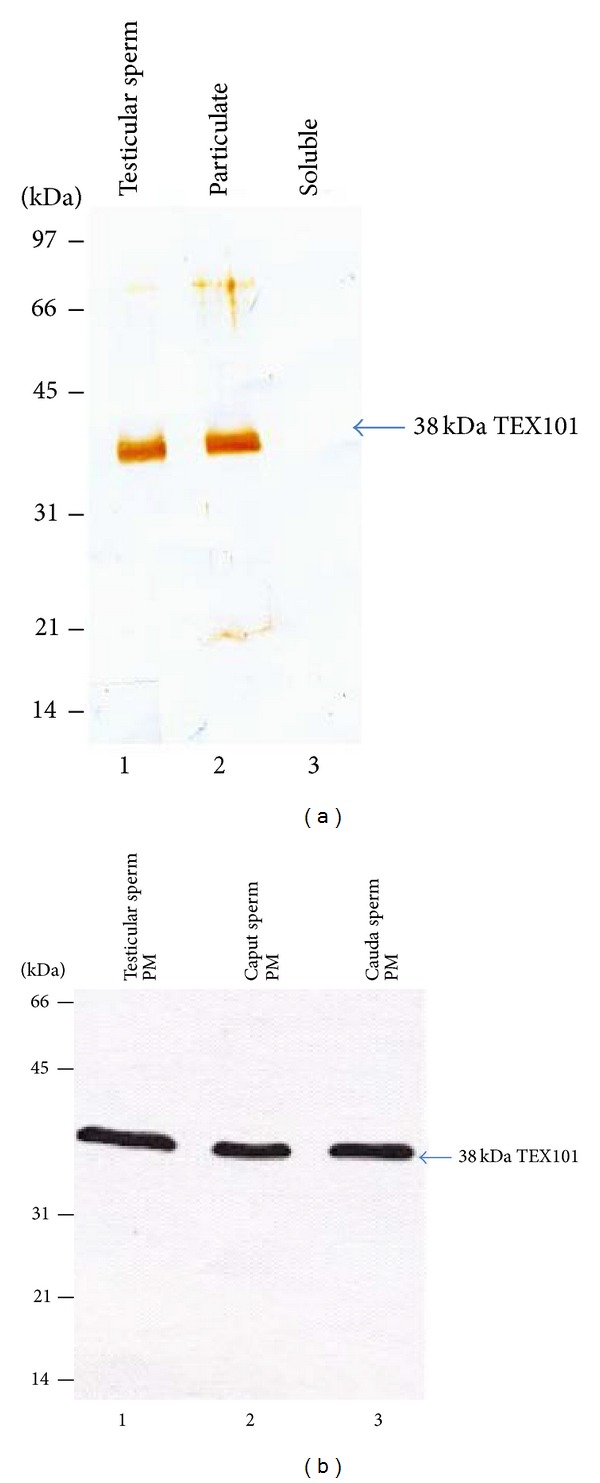
(a) Western blot immunostained with TEX101 antibody showing total bovine testicular sperm lysate (lane 1) and the particulate (lane 2) and soluble (lane 3) fractions obtained from testicular spermatozoa (2 × 10^6^ spermatozoa). TEX101 is only associated with the particulate fraction. (b) Immunoblot of plasma membrane fractions of testicular (lane 1), caput epididymal (lane 2), and cauda epididymal (lane 3) spermatozoa stained with anti-TEX101. Each lane contained 10 *μ*g protein. TEX101 is present in all three plasma membrane fractions.

Western blot analyses of the total (Figure 5(a), lane 1), the particulate (Figure 5(a), lane 2), and the soluble (Figure 5(a), lane 3) fractions of sonicated testicular spermatozoa stained with TEX 101 polyclonal antibody (1 : 1000 dilution) revealed that the 38 kDa TEX101 polypeptide was partitioned to the particulate fraction (lane 2). No immunoreactive band was seen when an identical blot was stained with TEX101 antibody preincubated with TEX101 (S-13) peptide (data is not shown) exhibiting the specificity of the TEX101 polyclonal antibody. This study reveals that TEX101 polypeptide is a membrane bound protein.

We next examined the biochemical localization of TEX101 in bovine testicular, caput, and cauda epididymal sperm plasma membrane fractions. Western blots of plasma membrane fractions of testicular (Figure 5(b), lane 1), caput (Figure 5(b), lane 2), and cauda (Figure 5(c), lane 3) epididymal sperm stained with TEX101 polyclonal antibody showed the presence of a 38 kDa TEX101 polypeptide in all plasma membrane fractions. An identical blot stained with nonimmune IgG showed no band (data is not shown). This study suggests that bovine TEX101 is present in both testicular and epididymal sperm plasma membrane fractions.

### 3.5. Immunolocalization of the TEX101 Protein

**Figure 6:**
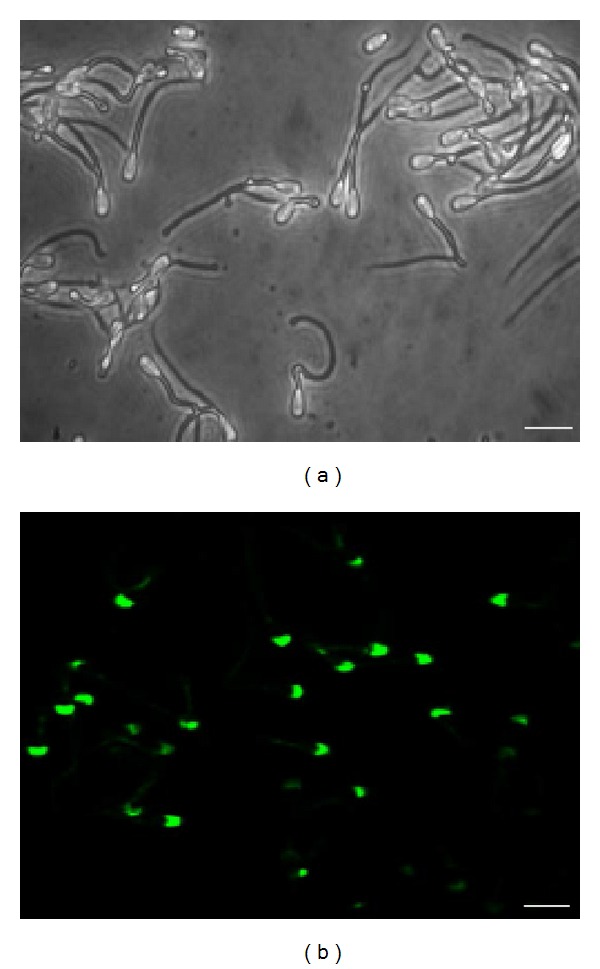
Immunofluorescence localization of the TEX101 protein. Matched phase contrast (a) and fluorescence (b) photomicrographs of permeabilized bovine cauda spermatozoa immunostained with anti-TEX101 polyclonal antibody. Staining was present on the head. Bar represents 10 *μ*m.

Light microscopic immunocytochemistry was utilized to define the localization of TEX101 polypeptide. Acetone permeabilized sperm exhibited intense staining on sperm head with anti-TEX101 (Figures 6(a) and 6(b)). No staining of the equatorial segment or of the postacrosomal segment of the head was noted. Control specimens of acetone permeabilized spermatozoa that were immunostained with identical dilution of antibody preincubated with TEX101 (S-13) peptide were completely negative (data is not in the text).

### 3.6. Evidence That Bovine Cauda Sperm Plasma Membrane TEX101 Is a Glycoprotein and Is a Glycosylphosphatidylinositol- (GPI-) Anchored Protein

**Figure 7:**
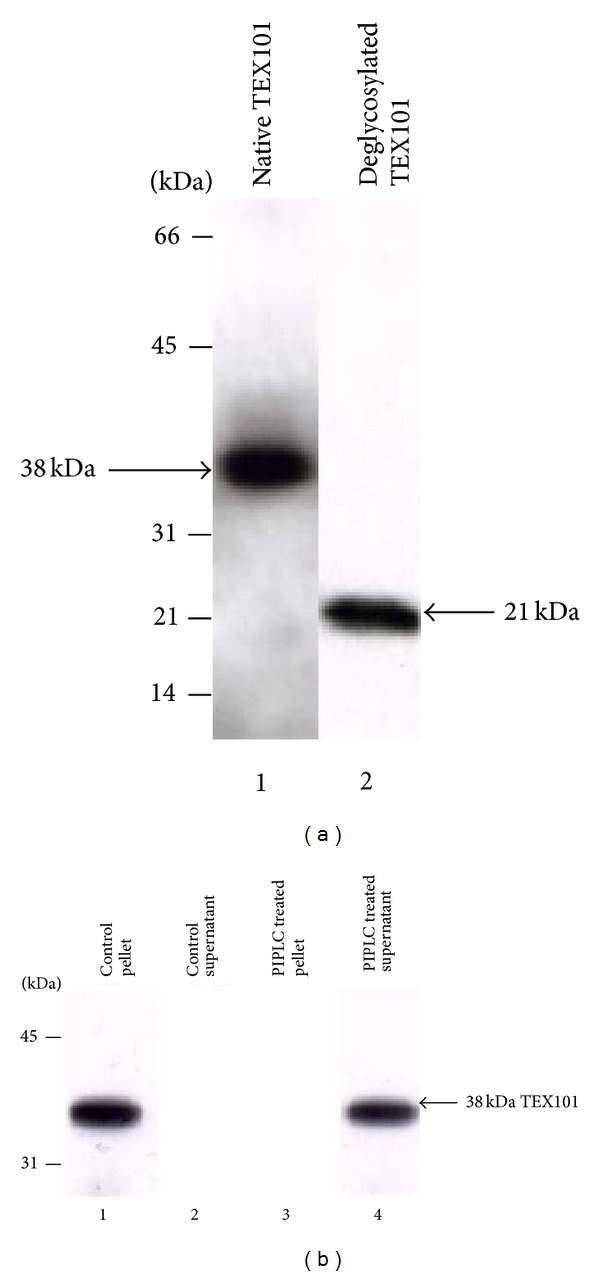
(a) Immunoblots of cauda sperm plasma membrane fraction treated with N-glycanase analyzed by reducing SDS-PAGE on 15% gels and immunostained with anti-TEX101. Lane 1 represents the untreated TEX101 polypeptide, and lane 2 displays N-glycanase treated TEX101 polypeptide. Note the reduction in molecular weight (~17 kDa) of the deglycosylated sample. Each lane contains 15 *μ*g protein. (b) Western blot analysis of TEX101 polypeptide treated with PIPLC and immunostained with anti-TEX101. Lanes 1 and 2 display the untreated plasma membranes (control) of pellet and supernatant fractions, respectively. TEX101 is present in the pellet fraction (lane 1). Lanes 3 and 4 exhibit the pellet and the supernatant fractions of PIPLC treated plasma membranes, respectively. Note that there is a complete release of TEX101 polypeptide in the supernatant fraction (lane 4) of PIPLC treated plasma membranes. The amount of plasma membrane proteins employed in the control and PIPLC treated experiments was 15 *μ*g.

To explore whether TEX101 is a glycoprotein, the cauda sperm plasma membranes were incubated in the absence or presence of N-glycanase, an endoenzyme that cleaves all N-linked oligosaccharide chains from glycoproteins [22]. Western blots of native TEX101 (Figure 7(a), lane 1) and N-linked deglycosylated TEX101 (Figure 7(a), lane 2) stained with TEX101 polyclonal antibody showed a significant reduction in the size of TEX101 polypeptide after N-glycanase treatment. Compared to native 38 kDa TEX101 polypeptide the deglycosylated form exhibited a 21 kDa single band. This study suggests that TEX101 possesses approximately 17 kDa of N-linked oligosaccharide moieties.

Attempts were then made to examine whether TEX101 is a GPI-anchored protein. Bovine cauda sperm plasma membrane incubated with PIPLC; PIPLC removes GPI-anchored proteins from the membrane [23]. Immunoblots of control (untreated plasma membrane) pellet revealed the presence of a 38 kDa TEX101 polypeptide (Figure 7(b), lane 1) whereas no release of TEX101 was observed in the control supernatant fraction (Figure 7(b), lane 2). On the contrary, after PIPLC treatment of plasma membrane, TEX101 polypeptide was completely released in the supernatant (Figure 7(b), lane 4). No TEX101 was detected in the PIPLC treated pellet fraction (Figure 7(b), lane 3). This experiment reveals that bovine cauda sperm TEX101 is anchored to membrane via a GPI-lipid linkage.

## 4. Discussion

Proteolytic processing by membrane-associated or luminal hydrolases and the binding of epididymal proteins are considered the primary mechanisms responsible for the modification of the sperm surface [1]. Our current study is focused on the identification and characterization of a 38 kDa RCA binding glycoprotein that is present in bovine caput and cauda epididymal sperm plasma membranes. Our data also reveals that the 38 kDa RCA binding polypeptide is not secreted by the epididymal epithelium. In the current study, we found that the 38 kDa polypeptide exhibits the highest affinity to RCA among the several major RCA binding polypeptides present in both caput and cauda sperm plasma membranes. Proteomic analysis of the purified 38 kDa RCA binding polypeptide matched the NCBI database sequence of TEX101 polypeptide. The predicted isoelectric point of mouse testicular TEX101 is 5.25 [24]. Our 2D-PAGE reveals a charge variant pattern of 38 kDa RCA binding polypeptide (TEX101) with three isoforms (pI 5.3–5.8) that may represent glycosylation variants. Functionally, it will be crucial to determine if the different TEX101 isoforms exhibit similar or different ligand-binding specificity.

Kurita et al. [24] first identified and characterized a 38 kDa TEX101 polypeptide in adult mouse testis and localized TEX101 on the plasma membrane of spermatocytes and spermatids. Biochemical localization of TEX101 in mouse testis homogenate revealed the presence of TEX101 in both the cytosol and detergent-soluble fractions [24]. However, our data showed the presence of TEX101 in the particulate fraction of bovine testicular spermatozoa (Figure 5(a)). In mouse, Takayama et al. [25] reported that TEX101 polypeptide is mostly removed from the surface of sperm during epididymal transit. On the contrary, Yin et al. [23] showed the presence of NYD-SP8 (homologous to mouse TEX101 polypeptide) in both human and mouse testes and human ejaculated and mouse cauda sperm. TEX101 was present in lipid raft fractions of mouse cauda epididymal sperm and the redistribution of TEX101 in lipid raft fractions occurs during capacitation [26]. Taken together, our studies strongly reveal the presence of TEX101 polypeptide in bovine cauda epididymal sperm plasma membranes.

Immunohistochemical analysis of TEX101 showed the presence of the polypeptide in the flagellum of mouse testicular sperm, whereas sperm in caput and corpus epididymis showed faint staining and no staining was observed in cauda sperm [25]. Miranda et al. [27] localized mouse cauda sperm TEX101 in the cytoplasmic droplet using the antibody provided by Kurita et al. [24]. The cytoplasmic droplet is generally regarded as a nonfunctional organelle containing residual cytoplasm which separates from the spermatozoon following its migration to the posterior end of the midpiece. Interestingly, the immunolocalization studies of Yin et al. [23] showed the presence of TEX101 to the posterior head of both human and mouse sperm, a region primarily involved in sperm-egg interaction. Our data also showed the presence of TEX101 protein in bovine cauda epididymal sperm head suggesting that the TEX101 glycocalyx may be involved in sperm-egg interaction. Due to the discrepancy of localization of TEX101 from the studies of several groups of investigators, the precise mechanism(s) of TEX101 polypeptide is not well defined. We will utilize electron microscopic immunocytochemical study in future to address this issue.

Mouse TEX is highly N-glycosylated [28] whereas bovine TEX101 contains 17 kDa N-linked oligosaccharides. The sugar moieties of TEX101 polypeptide may have a receptor-like role in sperm-egg interactions by recognizing its complementary molecule(s). Our future study will address the potential function of sugar moieties of TEX101 in fertilization process. Bovine cauda sperm TEX101 is anchored to the membrane via a GPI-lipid linkage (Figure 7(b), lane 4) as it was reported in mouse and human sperm TEX101 [23] and mouse testis TEX101 [28]. Immunoprecipitation, LC-MS-MS proteomic analysis, and immunofluorescence studies revealed the association of mouse testis TEX101 with cellubrevin [29], lymphocyte antigen 6 complex locus K (Ly6 K) [30, 31], and dipeptidase [32]. However, the precise functions of interactions of TEX101 to these three proteins are still unclear. Our future study will address the protein-protein interaction of bovine sperm TEX101 and the functional role of interaction either in sperm development or in fertilization process.

In sperm, it has been documented that the cleavage of GPI-anchored proteins is involved in fertilization [33]. Yin et al. [23] proposed that the sperm TEX101 may be involved in communicating with the cumulus of egg during sperm-egg interaction. Recently, it has also been shown that Tex101 gene knockout mice produce morphologically distinct spermatozoa but they do not have fertilization competency. Several members of cell adhesion proteins such as disintegrin and metallopeptidase domain 3 (ADAM3) were absent in cauda plasma membranes of Tex101^−/−^ mice suggesting that ADAM3 plays a significant role in causing the infertile phenotypes. They also observed that the existence of TEX101 on spermatozoa is regulated by angiotensin-converting enzyme (ACE) and to produce functionally competent spermatozoa, the removal of GPI-anchored TEX101 protein is required by ACE [34]. Another group of investigators showed that Tex101^−/−^ mouse sperm lost the adhesive competency to the surface of female genital tract. They also identified the loss of four ADAM proteins (ADAM3, ADAM4, ADAM5, and ADAM6) in Tex101^−/−^ mouse cauda epididymal spermatozoa suggesting that not only ADAM3 but also other ADAM protein family members might have an important role in sperm functions [35]. They also demonstrated that the infertility of TEX101^−/−^ mice is due to the uterotubal junction migration defect of sperm; however, TEX101^−/−^ sperm can fertilize oocytes both in vivo and in vitro via assisted reproduction. Additional studies are needed to localize TEX101 at the ultrastructural level and to resolve the discrepancy of physiological role of TEX101 in mammalian fertilization.

## 5. Conclusions

In the present study, we purified a 38 kDa* Ricinus Communis Agglutinin I* (RCA) binding glycoprotein present in bovine cauda epididymal sperm plasma membrane fractions having a charge train of three distinct spots with isoelectric points ranging between pH 5.3 and 5.8. Proteomic identification yielded ten peptides that matched the sequence of TEX101 (Bos Taurus). Our study reveals that TEX101 is present in both testicular and epididymal sperm plasma membrane fractions. Bovine cauda sperm TEX101 contains approximately 17 kDa of N-linked sugar residues and it is anchored to sperm membrane via a GPI-lipid linkage. Based on our biochemical data, we propose that bovine sperm TEX101 may play a significant role in fertilization event.

## References

[1] Yanagimachi R, Knobil E, Neill JD (1993). Mammalian fertilization. *The Physiology of Reproduction*.

[2] Eddy EM, O'Brien DA, Knobil E, Neill JD (1993). The spermatozoon. *The Physiology of Reproduction*.

[3] Eddy EM, O'Brien DA, Welch JE, Om W (1991). Mammalian sperm development in vivo and invitro. *Elements of Mammalian Fertilization*.

[4] Harayama H, Watanabe S-Y, Masuda H, Kannan Y, Miyake M, Kato S (1998). Lectin-binding characteristics of extracts from epididymal boar spermatozoa. *Journal of Reproduction and Development*.

[5] Magargee SF, Kunze E, Hammerstedt RH (1988). Changes in lectin-binding features of ram sperm surfaces associated with epididymal maturation and ejaculation. *Biology of Reproduction*.

[6] Mahmoud A, Parrish JJ (1996). Changes in lectin binding to bovine sperm during heparin-induced capacitation. *Molecular Reproduction Development*.

[7] Jones R (1998). Plasma membrane structure and remodelling during sperm maturation in the epididymis. *Journal of reproduction and fertility. Supplement*.

[8] Dacheux J-L, Belleannée C, Jones R (2009). Mammalian epididymal proteome. *Molecular and Cellular Endocrinology*.

[9] Aitken RJ, Nixon B, Lin M, Koppers AJ, Lee YH, Baker MA (2007). Proteomic changes in mammalian spermatozoa during epididymal maturation. *Asian Journal of Andrology*.

[10] Koehler JK (1981). Lectins as probes of the spermatozoon surface. *Archives of Andrology*.

[11] Young LG, Gould KG, Hinton BT (1986). Lectin binding sites on the plasma membrane of epididymal and ejaculated chimpanzee sperm. *Gamete Research*.

[12] Lee SH, Ahuja KK (1987). An investigation using lectins of glycocomponents of mouse spermatozoa during capacitation and sperm-zona binding. *Journal of Reproduction and Fertility*.

[13] Rankin TL, Holland ML, Orgebin-Crist M-C (1989). Lectin binding characteristics of mouse epididymal fluid and sperm extracts. *Gamete Research*.

[14] Srivastava A, Olson GE (1991). Glycoprotein changes in the rat sperm plasma membrane during maturation in the epididymis. *Molecular Reproduction and Development*.

[15] NagDas SK, Buchanan T, McCaskill S, Mackey J, Alvarez G, Raychoudhury S (2013). Identification of a calcium-binding protein of the acrsomal membrane of bovine spermatozoa. *The International Journal of Biochemistry and Cell Biology*.

[16] NagDas SK, Winfrey VP, Olson GE (2000). Identification of a hamster epididymal region-specific secretory glycoprotein that binds nonviable spermatozoa. *Biology of Reproduction*.

[17] NagDas SK, Winfrey VP, Olson GE (2002). Identification of Ras and its downstream signaling elements and their potential role in hamster sperm motility. *Biology of Reproduction*.

[18] Laemmli UK (1970). Cleavage of structural proteins during the assembly of the head of bacteriophage T4. *Nature*.

[19] Fairbanks G, Steck TL, Wallach DFH (1971). Electrophoretic analysis of the major polypeptides of the human erythrocyte membrane. *Biochemistry*.

[20] Wray W, Boulikas T, Wray VP, Hancock R (1981). Silver staining of proteins in polyacrylamide gels. *Analytical Biochemistry*.

[21] Towbin H, Staehelin T, Gordon J (1979). Electrophoretic transfer of proteins from polyacrylamide gels to nitrocellulose sheets: procedure and some applications. *Proceedings of the National Academy of Sciences of the United States of America*.

[22] Nagdas SK, Araki Y, Chayko CA, Orgebin-Crist M-C, Tulsiani DRP (1994). O-linked trisaccharide and N-linked poly-N-acetyllactosaminyl glycans are present on mouse ZP2 and ZP3. *Biology of Reproduction*.

[23] Yin L, Chung CM, Huo R (2009). A sperm GPI-anchored protein elicits sperm-cumulus cross-talk leading to the acrosome reaction. *Cellular and Molecular Life Sciences*.

[24] Kurita A, Takizawa T, Takayama T (2001). Identification, cloning, and initial characterization of a novel mouse testicular germ cell-specific antigen. *Biology of Reproduction*.

[25] Takayama T, Mishima T, Mori M (2005). TEX101 is shed from the surface of sperm located in the caput epididymidis of the mouse. *Zygote*.

[26] Sleight SB, Miranda PV, Plaskett N-W (2005). Isolation and proteomic analysis of mouse sperm detergent-resistant membrane fractions: evidence for dissociation of lipid rafts during capacitation. *Biology of Reproduction*.

[27] Miranda PV, Allaire A, Sosnik J, Visconti PE (2009). Localization of low-density detergent-resistant membrane proteins in intact and acrosome-reacted mouse sperm. *Biology of Reproduction*.

[28] Jin H, Yoshitake H, Tsukamoto H (2006). Molecular characterization of a germ-cell-specific antigen, TEX101, from mouse testis. *Zygote*.

[29] Tsukamoto H, Yoshitake H, Mori M (2006). Testicular proteins associated with the germ cell-marker, TEX101: involvement of cellubrevin in TEX101-trafficking to the cell surface during spermatogenesis. *Biochemical and Biophysical Research Communications*.

[30] Yoshitake H, Tsukamoto H, Maruyama-Fukushima M, Takamori K, Ogawa H, Araki Y (2008). TEX101, a germ cell-marker glycoprotein, is associated with lymphocyte antigen 6 complex locus k within the mouse testis. *Biochemical and Biophysical Research Communications*.

[31] Maruyama M, Yoshitake H, Tsukamoto H, Takamori K, Araki Y (2010). Molecular expression of Ly6k, a putative glycosylphosphatidyl-inositol-anchored membrane protein on the mouse testicular germ cells. *Biochemical and Biophysical Research Communications*.

[32] Yoshitake H, Yanagida M, Maruyama M, Takamori K, Hasegawa A, Araki Y (2011). Molecular characterization and expression of dipeptidase 3, a testis-specific membrane-bound dipeptidase: complex formation with TEX101, a germ-cell-specific antigen in the mouse testis. *Journal of Reproductive Immunology*.

[33] Kondoh G, Tojo H, Nakatani Y (2005). Angiotensin-converting enzyme is a GPI-anchored protein releasing factor crucial for fertilization. *Nature Medicine*.

[34] Fujihara Y, Tokuhiro K, Muro Y (2013). Expression of TEX101, regulated by ACE, is essential for the production of fertile mouse spermatozoa. *Proceedings of the National Academy of Sciences of the United States of America*.

[35] Li W, Guo XJ, Teng F (2013). Tex101 is essential for male fertility by affecting sperm migration into the oviduct in mice. *Journal of Molecular Cell Biology*.

